# Network Pharmacology and In Vivo Validation Reveal Berberine-Mediated Regulation of the Liver–Brain Inflammatory Axis in MCD-Induced Steatohepatitis

**DOI:** 10.3390/ijms27156967

**Published:** 2026-08-03

**Authors:** Yeon-Joo Yoo, Ji-Han Kim, Seung-Hoon Yoo, Byung-Cheol Lee

**Affiliations:** 1 Department of Clinical Korean Medicine, Graduate School, Kyung Hee University, 26, Kyungheedae-ro, Dongdaemun-gu, Seoul 02447, Republic of Korea; alice98113@khu.ac.kr (Y.-J.Y.); ilpc21@khu.ac.kr (J.-H.K.); ysh9306@khu.ac.kr (S.-H.Y.); 2 Department of Nephrology & Endocrinology, Kyung Hee University Korean Medicine Hospital, Kyung Hee Medical Center, 23 Kyungheedae-ro, Dongdaemun-gu, Seoul 02447, Republic of Korea

**Keywords:** berberine, *Coptidis Rhizoma*, metabolic dysfunction-associated steatohepatitis (MASH), liver–brain axis, network pharmacology, molecular docking

## Abstract

Metabolic dysfunction-associated steatohepatitis (MASH) is a progressive immunometabolic liver disorder involving lipid dysregulation, inflammation, fibrosis, and extrahepatic immune–neural responses, yet therapies capable of modulating these interconnected processes remain limited. Berberine (BBR), an isoquinoline alkaloid derived from traditional medicinal plants including *Coptis chinensis* Franch. (Coptidis Rhizoma), has shown metabolic and anti-inflammatory activities; however, its effects on hepatic inflammation and the liver–brain inflammatory axis in MASH remain unclear. Here, network pharmacology and molecular docking were used to predict BBR targets and pathways, followed by in vivo validation in a methionine- and choline-deficient diet-induced mouse model. Liver injury and metabolic alterations were assessed using serum biochemistry and lipid profiles, histological changes by hematoxylin and eosin and Sirius Red staining, and hepatic and hypothalamic inflammation by qRT-PCR, flow cytometry, and Iba-1/GFAP immunostaining. SREBF1, AKT1, and TGFB1 were identified as core BBR targets, with pathways linked to lipid metabolism, oxidative stress, inflammation, and fibrogenesis. BBR attenuated liver injury, steatosis, steatohepatitis, and fibrosis, suppressed SREBF1-associated lipogenic signaling and fibrogenic gene expression, remodeled circulating monocyte subsets, reduced Kupffer cell accumulation, and inhibited hypothalamic microglial activation. These findings suggest that BBR alleviates MCD-induced steatohepatitis through multi-target regulation of hepatic metabolic dysfunction, immune remodeling, and hypothalamic neuroinflammation.

## 1. Introduction

Metabolic dysfunction-associated steatohepatitis (MASH) is a progressive liver disorder characterized by hepatic lipid accumulation, hepatocellular injury, inflammation and fibrosis, resulting from the complex interaction between metabolic stress and immune dysregulation [1]. The liver continuously maintains metabolic homeostasis through coordinated regulation of lipid metabolism, mitochondrial function, oxidative stress responses, and inflammatory signaling pathways [2]. However, excessive lipid accumulation and lipotoxicity disrupt this delicate balance and trigger a pathological cascade involving oxidative stress, Kupffer cell activation, inflammatory cell infiltration, and fibrogenic remodeling [3]. Therefore, restoration of hepatic metabolic and inflammatory homeostasis remains highly challenging and is frequently complicated by persistent MASH, progressive fibrosis, and extrahepatic inflammatory responses [4]. With the increasing prevalence of obesity, insulin resistance, and metabolic syndrome, MASH has become a major cause of chronic liver disease worldwide and represents a growing clinical and socioeconomic burden [5]. Current therapeutic management of MASH primarily depends on lifestyle modification, including dietary control, weight reduction, and exercise, which can improve metabolic parameters but are difficult to maintain in the long term [6]. Although several pharmacological agents targeting lipid metabolism, insulin resistance, inflammation, and fibrosis have been investigated, effective treatment options that simultaneously modulate the multiple pathological processes of MASH remain limited [7].

In particular, hepatic inflammation and fibrosis are driven by complex interactions among hepatocytes, Kupffer cells, recruited monocytes, hepatic stellate cells, and systemic immune mediators [8]. However, most current therapeutic approaches focus on single targets, which may explain their limited clinical efficacy [9]. Recent evidence also suggests that metabolic liver disease may influence central inflammatory responses through liver–brain communication [10], including hypothalamic microglial activation and neuroinflammation [11]. Therefore, novel therapeutic strategies capable of regulating hepatic metabolic dysfunction, immune cell remodeling, fibrogenesis, and extrahepatic inflammatory communication are required.

Natural products have emerged as promising candidates for MASH because of their multitarget bioactive properties, including lipid-lowering, anti-inflammatory, antioxidant, and antifibrotic effects [12]. In traditional medicine research, various medicinal herbs and plant-derived compounds have been studied for their ability to regulate hepatic lipid metabolism, suppress inflammatory signaling, and attenuate liver fibrosis [13]. However, there remains a need to identify potent candidates that can simultaneously modulate these responses associated with MASH.

Berberine (BBR) is a major isoquinoline alkaloid found in several traditional medicinal plants, including *Coptis chinensis* Franch. (*Coptidis Rhizoma*, Huanglian), *Phellodendron amurense* Rupr. (*Phellodendri Cortex*, Huangbai), and Berberis species. *Coptidis Rhizoma* has been traditionally used to clear heat, dry dampness, purge fire, and detoxify, and its traditional applications include damp-heat-related gastrointestinal disorders, jaundice, and diabetes [14]. Previous studies have reported that BBR exhibits diverse pharmacological activities, including regulation of glucose and lipid metabolism, suppression of oxidative stress, inhibition of inflammatory cytokine production, and attenuation of fibrosis-related signaling [15]. Given that MASH involves hepatic lipid accumulation, inflammatory injury, fibrosis, and systemic metabolic dysregulation, BBR is a highly plausible candidate for modulating this immunometabolic pathology.

However, most previous studies have relied on high-fat diet models that do not fully recapitulate the inflammatory and fibrotic features of advanced MASH [16], and its effects on systemic immunometabolic regulation and central neuroinflammation remain unclear. Therefore, we evaluated the therapeutic potential of BBR using a methionine- and choline-deficient diet-induced mouse model of steatohepatitis. Network pharmacology analysis was conducted to predict the potential molecular targets and signaling pathways associated with the effects of BBR, followed by molecular docking and in vivo validation. Our findings provide mechanistic evidence that BBR attenuates steatohepatitis and suggest that its therapeutic effects may involve coordinated regulation of hepatic lipogenic, inflammatory, and fibrogenic responses, as well as modulation of the liver–brain inflammatory axis.

## 2. Results

### 2.1. Network Pharmacology and Molecular Docking Predicted Potential Mechanisms of BBR in MASH

Recently, in silico approaches, including ADME (absorption, distribution, metabolism, and excretion) prediction, network pharmacology, and molecular docking, have been widely used to identify bioactive compounds, therapeutic targets, and key regulatory pathways involved in complex metabolic diseases. Among the screened compounds, berberine (BBR) was selected for further investigation based on its well-established pharmacological relevance to metabolic and inflammatory processes implicated in MASH. To elucidate the molecular basis of BBR in metabolic dysfunction-associated steatohepatitis (MASH), network pharmacology analysis was performed to identify BBR-associated targets and MASH-related genes. A total of 357 BBR target genes and 1028 MASH-associated genes were collected, among which 156 overlapping targets were identified as putative therapeutic targets of BBR in MASH.

Functional enrichment analysis demonstrated that these overlapping targets were significantly involved in biological processes associated with lipid metabolism, inflammatory signaling, fibrogenesis, metabolic regulation, cellular stress, and apoptosis. KEGG pathway enrichment further highlighted several mechanistic pathways closely related to MASH pathogenesis, suggesting that BBR may exert therapeutic effects through coordinated regulation of metabolic, inflammatory, and fibrogenic networks. To further explore the functional interactions among these targets, a protein–protein interaction (PPI) network was constructed using the STRING database. The PPI network showed a highly interconnected architecture, indicating that BBR may modulate MASH through multi-target regulatory mechanisms rather than through a single molecular target. Based on network topology and functional relevance, key hub genes were prioritized for subsequent pathway interpretation and molecular docking analysis.

Representative targets involved in lipid uptake and metabolic regulation, including CD36, FGF21, INS, LEP, PNPLA3, PPARγ, and SREBF1, were selected for docking analysis. In addition, targets associated with inflammatory and fibrogenic signaling, including CCL2, IL6, TNF-α, MAPK8, TGFB1, TNFRSF1A, and TRAF2, as well as cellular stress- and apoptosis-related targets, including AKT1, BCL2L11, CASP3, CASP8, CYCS, and JUN, were further evaluated. Molecular docking was performed to validate the potential interactions between BBR and these core regulatory targets identified from the network pharmacology analysis.

**Figure 1:**
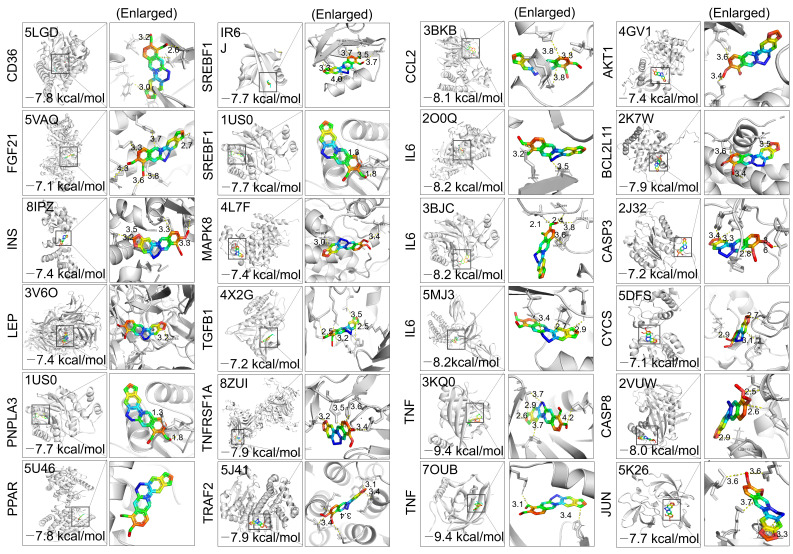
BBR exhibits strong binding affinity to key regulatory targets involved in MASH-related inflammatory and fibrotic pathology. Representative molecular docking conformations showing the overall binding pose of Berberine with target proteins (**left panel**) and a magnified view of the key interaction sites (**right panel**). Docked targets categorized according to their biological functions in MASH, including lipid metabolism (CD36, CD36 molecule; FGF21, fibroblast growth factor 21; INS, insulin; LEP, leptin; PNPLA3, patatin-like phospholipase domain-containing protein 3; PPARγ, peroxisome proliferator-activated receptor; SREBF1, sterol regulatory element-binding transcription factor 1), fibrosis (MAPK8, mitogen-activated protein kinase 8; TGFB1, transforming growth factor beta 1; TNFRSF1A, tumor necrosis factor receptor superfamily member 1A; TRAF2, TNF receptor-associated factor 2), inflammation (CCL2, chemokine [C–C motif] ligand 2; IL6, interleukin-6; TNF-α, tumor necrosis factor alpha), metabolic regulation (AKT1, AKT serine/threonine kinase 1; FGF21), and apoptosis (BCL2L11, BCL2-like 11; CASP3, caspase 3; CASP8, caspase 8; CYCS, cytochrome c; JUN, Jun proto-oncogene, AP-1 transcription factor subunit).

As summarized in Figure 1, BBR exhibited favorable binding affinities toward multiple key targets, with binding energies ranging from −7.1 to −9.4 kcal/mol. Among the docked targets, strong binding affinities were observed for inflammatory mediators, including TNF-α, IL6, and CCL2, as well as apoptosis- and fibrosis-related targets such as CASP3, TNFRSF1A, and BCL2L11. BBR also showed favorable interactions with major regulators of lipid metabolism and metabolic signaling, including CD36, PPARγ, PNPLA3, INS, LEP, FGF21, and SREBF1. In addition, BBR interacted with targets involved in fibrogenic and cellular stress pathways, including MAPK8, TGFB1, TRAF2, CASP8, CYCS, and JUN. Notably, SREBF1, AKT1, and TGFB1 were identified as key regulatory nodes that mechanistically link metabolic dysregulation, inflammatory activation, and fibrogenic remodeling in MASH. These findings suggest that the protective effects of BBR against MASH may be mediated by multi-target binding and coordinated regulation of lipid metabolic, inflammatory, apoptotic, and fibrogenic pathways.

### 2.2. BBR Did Not Prevent MCD-Induced Body Weight Loss Despite Increased Caloric Intake

**Figure 2:**
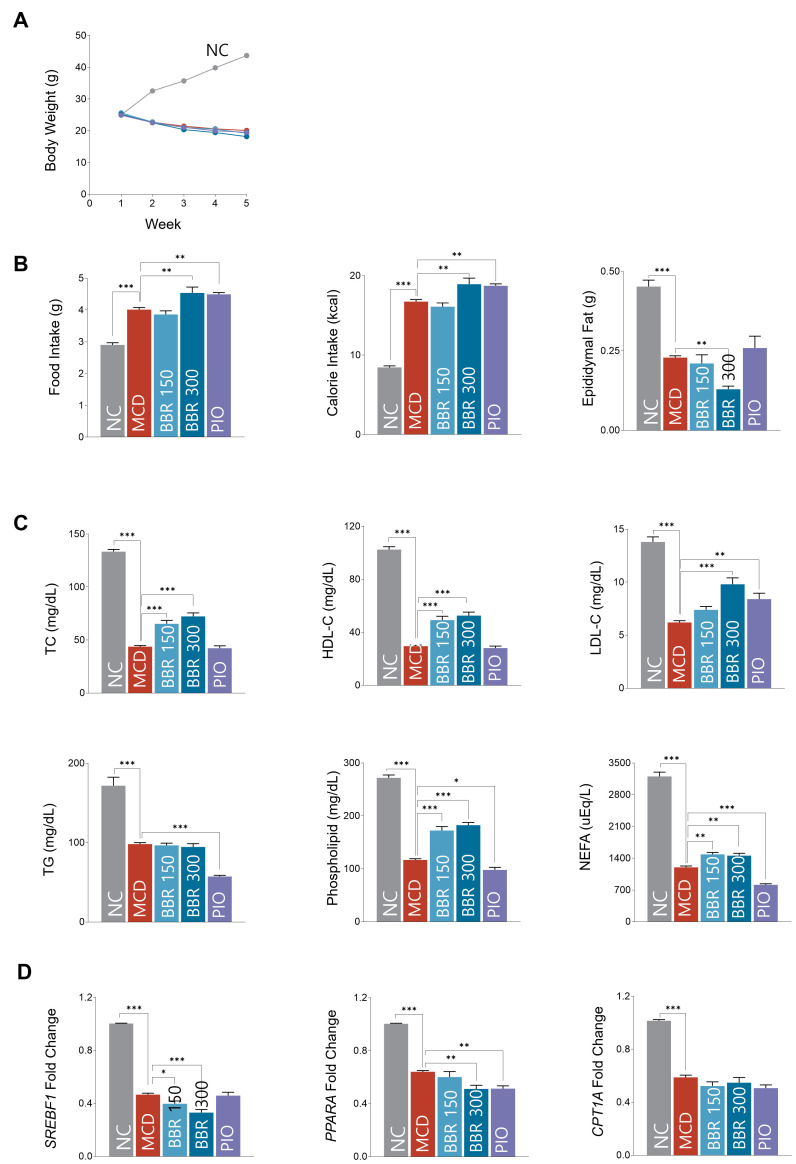
BBR regulates lipid metabolic pathways in MCD diet-induced MASH. (**A**) BBR has no effect on body weight change. (**B**) BBR changes food and calorie intake; quantitative analysis of epididymal fat weight in the indicated groups. (**C**) Serum lipid profile parameters. (**D**) Relative mRNA expression levels of genes associated with lipid metabolism, including SREBF1 (sterol regulatory element-binding transcription factor 1), FASN (fatty acid synthase), PPARα (peroxisome proliferator-activated receptor alpha), and CPT1A (carnitine palmitoyl transferase 1A) regulated by BBR. Data are presented as mean ± SEM; * *p* < 0.05, ** *p* < 0.01, *** *p* < 0.001; NC, Normal Chow group; MCD, MCD diet group; BBR 150, MCD diet + Berberine 150 mg/kg/day; BBR 300, MCD diet + Berberine 300 mg/kg/day; PIO, MCD diet + Pioglitazone 30 mg/kg/day.

To evaluate whether the effects of BBR were associated with changes in body weight or energy intake, body weight, daily food intake, and caloric intake were monitored during the experimental period (Figure 2A,B). At baseline, the mean body weight was comparable among the five groups, with values of 25.00 ± 0.32 g in the NC group, 25.40 ± 0.40 g in the MCD(methionine- and choline-deficient) group, 25.64 ± 0.68 g in the BBR 150 group, 25.26 ± 0.16 g in the BBR 300 group, and 24.94 ± 0.47 g in the PIO group.

After 4 weeks, the NC group showed a mean body weight gain of 4.98 ± 0.52 g, whereas the MCD group exhibited a significant reduction in body weight compared with the NC group (−4.90 ± 0.24 g, *p* < 0.001), consistent with the catabolic phenotype of the MCD diet model. Similarly, BBR-treated mice showed body weight reduction, with mean changes of −6.40 ± 0.94 g in the BBR 150 group and −7.08 ± 0.68 g in the BBR 300 group. However, these changes were not statistically significant compared with the MCD group.

Daily food intake was significantly increased in the MCD group compared with the NC group (4.01 ± 0.20 g vs. 2.90 ± 0.15 g, *p* < 0.001). Furthermore, food intake was markedly elevated in the BBR 300 and PIO groups compared with the MCD group (4.54 ± 0.41 g and 4.49 ± 0.13 g, respectively; both *p* < 0.01). Caloric intake showed a similar pattern. The MCD group consumed significantly more calories than the NC group (16.73 ± 0.85 kcal vs. 8.45 ± 0.43 kcal, *p* < 0.001), and caloric intake was further increased in the BBR 300 and PIO groups compared with the MCD group (18.93 ± 1.70 kcal and 18.74 ± 0.52 kcal, respectively; both *p* < 0.01).

Collectively, these findings indicate that BBR did not prevent MCD diet-induced body weight loss despite increased food and caloric intake. The MCD diet induces profound body weight loss as a consequence of methionine and choline deficiency, resulting in systemic metabolic alterations that are largely independent of hepatic lipid metabolism. Therefore, the absence of differences in body weight among the MCD-fed groups is consistent with the intrinsic characteristics of the MCD model and does not necessarily reflect the efficacy of BBR in improving hepatic pathology.

### 2.3. BBR Restored Lipid Homeostasis Through Suppression of the Srebf1-Centered Lipogenic Axis

To determine the effects of BBR on lipid metabolic regulation in MCD diet-induced MASH, adipose tissue mass and hepatic lipid-related gene expression were evaluated at week 5. At this time point, epididymal fat pad weight was significantly reduced in the MCD group compared with the NC group (0.23 ± 0.02 g vs. 0.45 ± 0.04 g, *p* < 0.001), reflecting marked adipose tissue loss associated with the catabolic phenotype of the MCD diet model. Although adipose tissue mass was already decreased by MCD feeding, BBR administration further reduced epididymal fat pad weight, with the lowest value observed in the BBR 300 group compared with the MCD group (0.13 ± 0.02 g vs. 0.23 ± 0.02 g, *p* < 0.01) (Figure 2C). In contrast, pioglitazone-treated mice exhibited relatively higher epididymal fat pad weight than BBR-treated mice.

To further clarify whether these changes were associated with hepatic lipid metabolic regulation, the expression of lipid-related genes was analyzed. Quantitative analysis confirmed that BBR significantly suppressed hepatic SREBF1 expression in a dose-dependent manner, decreasing from 0.47 ± 0.03 in the MCD group to 0.40 ± 0.05 in the BBR 150 group (*p* < 0.05) and 0.33 ± 0.05 in the BBR 300 group (*p* < 0.001). CPT1A expression also showed a decreasing tendency following BBR treatment, although this change was not statistically significant (Figure 2D). These results indicate that BBR primarily influenced hepatic lipogenic regulation rather than preventing MCD-induced adipose tissue loss.

Collectively, these findings suggest that BBR modulated lipid metabolic responses in MCD diet-induced steatohepatitis, at least in part through suppression of the SREBF1-associated lipogenic axis. Since adipose tissue depletion is a characteristic feature of the MCD diet model, the lipid-regulatory effects of BBR were interpreted mainly based on hepatic lipogenic gene expression rather than restoration of peripheral adiposity.

### 2.4. BBR Suppressed MASH and Fibrogenic Responses

**Figure 3:**
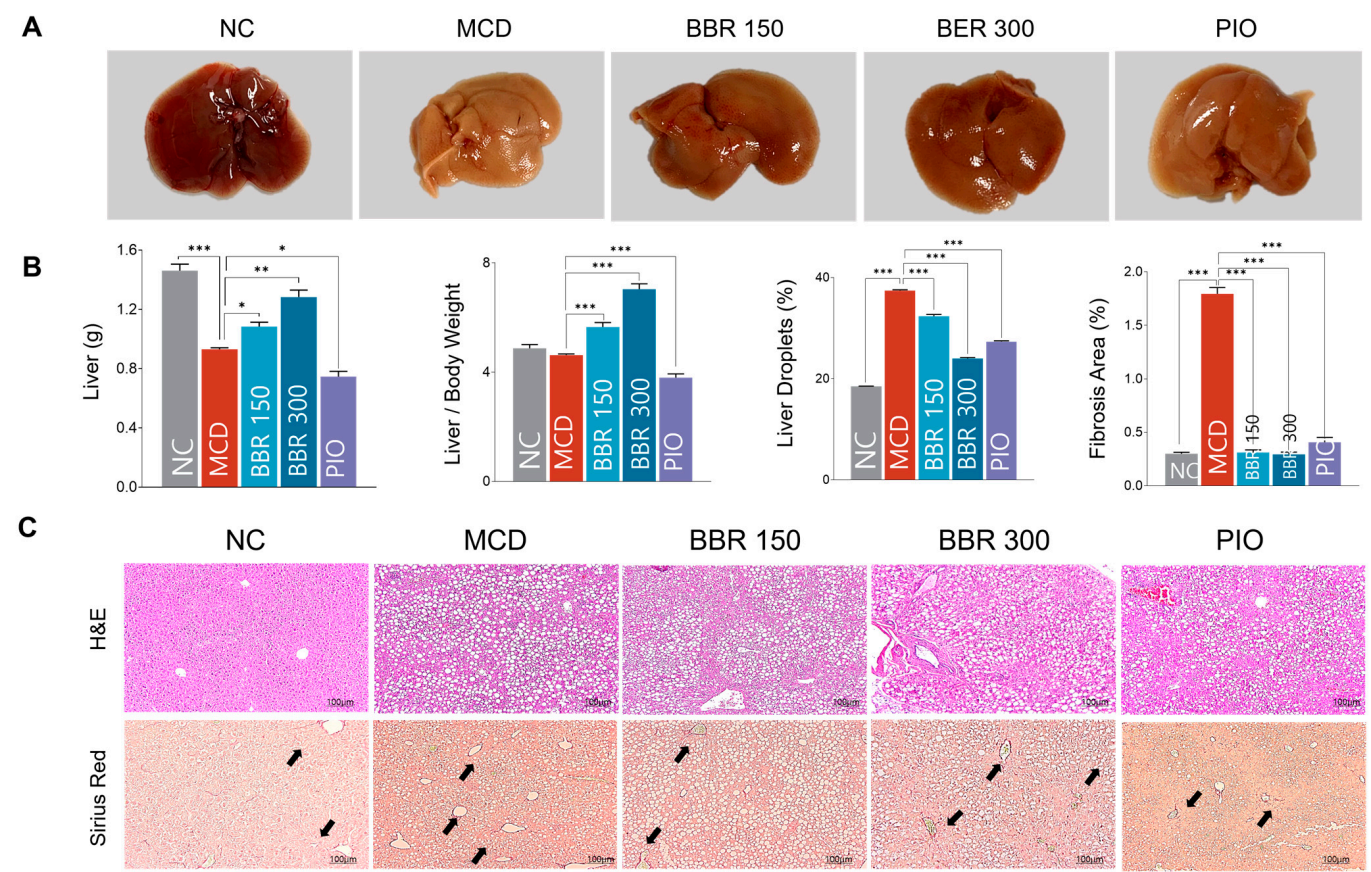
BBR attenuates hepatic steatosis, fibrosis, and functional injury in MCD diet-induced MASH. (**A**) Representative images of gross liver morphology in the indicated groups. (**B**) Quantitative analysis of liver weight, liver-to-body weight ratio, hepatic lipid droplet accumulation and fibrotic area. (**C**) Representative histological images of liver sections stained with hematoxylin and eosin (H&E) and Sirius Red. Data are presented as mean ± SEM; * *p* < 0.05, ** *p* < 0.01, *** *p* < 0.001; NC, Normal Chow group; MCD, MCD diet group; BBR 150, MCD diet + Berberine 150 mg/kg/day; BBR 300, MCD diet + Berberine 300 mg/kg/day; PIO, MCD diet + Pioglitazone 30 mg/kg/day. Arrows indicate hepatic lipid droplet area.

To evaluate the effects of BBR on hepatic injury and structural alterations in MCD diet-induced steatohepatitis, liver weight, gross morphology, histological changes, serum biochemical markers, and fibrosis-related gene expression were analyzed. MCD feeding markedly reduced liver weight compared with the NC group (0.93 ± 0.03 g vs. 1.46 ± 0.10 g, *p* < 0.001), reflecting severe hepatic injury and tissue atrophy. In contrast, BBR treatment significantly increased liver weight in a dose-dependent manner, with recovery observed in both the BBR 150 group (1.08 ± 0.07 g vs. 0.93 ± 0.03 g, *p* < 0.05) and the BBR 300 group (1.28 ± 0.10 g vs. 0.93 ± 0.03 g, *p* < 0.01) compared with the MCD group. The liver-to-body weight ratio showed a similar increasing pattern in the BBR 150 and BBR 300 groups compared with the MCD group (5.66 ± 0.36 and 7.04 ± 0.44 vs. 4.63 ± 0.13, respectively; both *p* < 0.001), whereas pioglitazone significantly decreased this ratio (Figure 3A,B). Gross liver morphology further supported these findings, showing prominent liver size reduction in the MCD group and partial restoration in the BBR-treated groups.

Histological examination using H&E staining revealed no apparent hepatic abnormalities in the NC group, whereas MCD-fed mice exhibited extensive hepatocellular vacuolation and lipid droplet accumulation. Quantitative analysis confirmed that hepatic lipid deposition was significantly increased in the MCD group compared with the NC group. BBR treatment markedly reduced hepatic lipid accumulation, with significant decreases in both the BBR 150 group (32.35 ± 0.81% vs. 37.42 ± 0.38%, *p* < 0.001) and the BBR 300 group (24.01 ± 0.39% vs. 37.42 ± 0.38%, *p* < 0.001) compared with the MCD group. Pioglitazone also reduced lipid deposition; however, this effect was less pronounced than that observed in the high-dose BBR group. Sirius Red staining further demonstrated that MCD feeding induced marked hepatic collagen deposition compared with the NC group. Conversely, BBR administration substantially attenuated this fibrotic response, with fibrosis areas reduced to 0.31 ± 0.05% in the BBR 150 group and 0.30 ± 0.05% in the BBR 300 group, both of which were significantly lower than those in the MCD group (*p* < 0.001) and comparable to the NC group (Figure 3A–C). Pioglitazone also reduced MCD-induced fibrosis, although the effect was numerically weaker than that of BBR.

**Figure 4:**
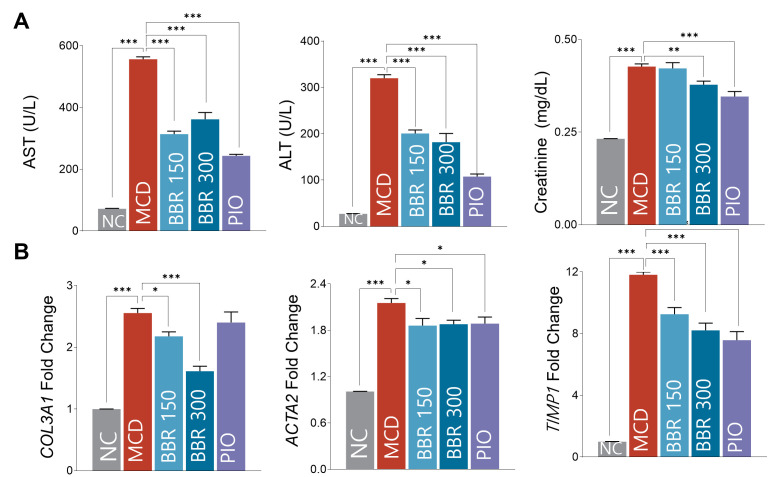
BBR attenuates liver injury and fibrosis-related gene expression. (**A**) Serum biochemical parameters reflecting liver function. (**B**) Relative mRNA expression levels of fibrosis-related genes, including COL3A1 (collagen type III alpha 1 chain), ACTA2 (actin alpha 2), and TIMP1 (TIMP metallopeptidase inhibitor 1). Data are presented as mean ± SEM; * *p* < 0.05, ** *p* < 0.01, *** *p* < 0.001; NC, Normal Chow group; MCD, MCD diet group; BBR 150, MCD diet + Berberine 150 mg/kg/day; BBR 300, MCD diet + Berberine 300 mg/kg/day; PIO, MCD diet + Pioglitazone 30 mg/kg/day.

These structural and histological improvements were accompanied by amelioration of biochemical alterations, as demonstrated by significant reductions in serum AST, ALT, and creatinine levels following BBR treatment (Figure 4A). To further determine whether BBR regulated fibrogenic activation at the molecular level, the mRNA expression of fibrosis-related genes was examined. MCD feeding markedly upregulated COL3A1, ACTA2, TGFB1, and TIMP1 expression, whereas BBR treatment suppressed these fibrogenic markers, particularly COL3A1, ACTA2, and TIMP1. COL3A1 expression was reduced from 2.56 ± 0.24 in the MCD group to 2.18 ± 0.16 in the BBR 150 group (*p* < 0.05) and 1.60 ± 0.18 in the BBR 300 group (*p* < 0.001). ACTA2 expression was also decreased following BBR treatment, while TIMP1 expression was significantly reduced in both BBR-treated groups and the pioglitazone group (BBR 150, 9.26 ± 0.96; BBR 300, 8.21 ± 1.05; PIO, 7.58 ± 1.24 vs. MCD, 11.82 ± 0.57; all *p* < 0.001) (Figure 4B).

Collectively, these findings demonstrate that BBR attenuated MCD diet-induced MASH by reducing hepatic lipid accumulation, collagen deposition, biochemical liver injury, and fibrogenic gene expression. These results suggest that BBR exerts hepatoprotective effects by suppressing both steatotic and fibrogenic responses in MASH-like pathology.

### 2.5. BBR Modulated Systemic Immune Responses and Attenuated Hypothalamic Neuroinflammation

**Figure 5:**
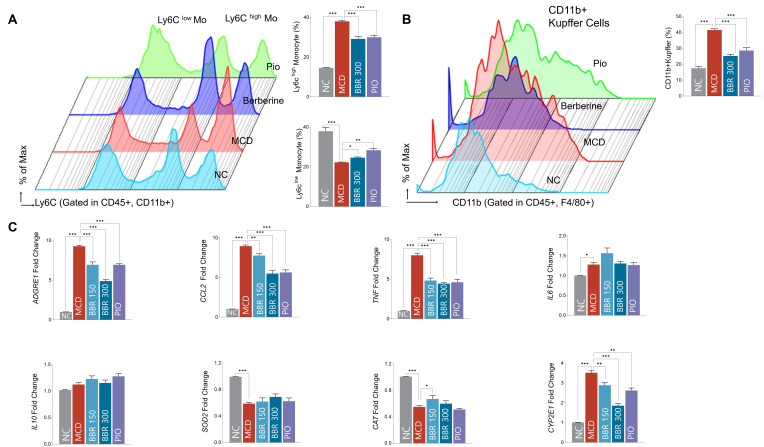
BBR exerts systemic immunomodulatory effects in MCD diet-induced MASH. (**A**) Representative flow cytometry gating strategy and quantitative analysis of circulating Ly6Chi and Ly6Clo monocyte populations in the indicated groups. (**B**) Representative gating and percentage of hepatic F4/80^+^ macrophage/Kupffer cell-enriched populations. (**C**) mRNA expression levels of genes associated with inflammation and oxidative stress including ADGRE1 (adhesion G protein-coupled receptor E1), CCL2 (chemokine [C–C motif] ligand 2), TNF-α (tumor necrosis factor alpha), IL6 (interleukin-6), IL10 (interleukin-10), SOD2 (superoxide dismutase 2), CAT (catalase), and CYP2E1 (cytochrome P450 family 2 subfamily E member 1). Data are presented as mean ± SEM; * *p* < 0.05, ** *p* < 0.01, *** *p* < 0.001; NC, Normal Chow group; MCD, MCD diet group; BBR 150, MCD diet + Berberine 150 mg/kg/day; BBR 300, MCD diet + Berberine 300 mg/kg/day; PIO, MCD diet + Pioglitazone 30 mg/kg/day.

To determine whether BBR regulates inflammatory and neuroimmune responses in MCD diet-induced steatohepatitis, circulating monocyte subsets, hepatic macrophage/Kupffer cell accumulation, inflammatory gene expression, and hypothalamic glial activation were analyzed. Flow cytometric analysis demonstrated that MCD feeding induced a marked systemic pro-inflammatory shift, as evidenced by a significant increase in Ly6Chi monocytes and a concomitant decrease in Ly6Clo monocytes compared with the NC group. In contrast, BBR treatment dose-dependently reduced the proportion of Ly6Chi monocytes, with values of 32.23 ± 3.07% in the BBR 150 group and 29.14 ± 2.53% in the BBR 300 group compared with the MCD group (both *p* < 0.001). In addition, BBR partially restored Ly6Clo monocytes in the BBR 300 group compared with the MCD group (24.59 ± 0.80% vs. 22.23 ± 0.58%, *p* < 0.05), suggesting a shift toward a less inflammatory and more resolving immune phenotype (Figure 5A).

Consistent with systemic immune activation, hepatic F4/80^+^ macrophage/Kupffer cell-enriched populations were significantly increased in the MCD group compared with the NC group. Conversely, BBR treatment dose-dependently attenuated this increase, with values of 30.55 ± 2.34% in the BBR 150 group and 25.17 ± 2.26% in the BBR 300 group compared with the MCD group (41.51 ± 2.66%; both *p* < 0.001) (Figure 5B). These findings indicate that BBR suppressed both circulating pro-inflammatory monocyte expansion and hepatic macrophage/Kupffer cell accumulation in MCD-fed mice.

To further evaluate whether these cellular changes were accompanied by molecular regulation of hepatic inflammation and oxidative stress, the expression of related markers was examined. BBR treatment significantly suppressed pro-inflammatory and macrophage-associated genes, including ADGRE1, CCL2, TNF-α, and IL6, with greater effects observed at the higher dose. ADGRE1 expression decreased from 9.27 ± 0.42 in the MCD group to 6.93 ± 0.81 in the BBR 150 group and 4.88 ± 0.45 in the BBR 300 group (both *p* < 0.001). CCL2 expression was also reduced from 8.95 ± 0.44 in the MCD group to 7.76 ± 0.57 in the BBR 150 group (*p* < 0.01) and 5.49 ± 0.84 in the BBR 300 group (*p* < 0.001). Similarly, TNF-α expression decreased from 8.00 ± 0.84 in the MCD group to 4.82 ± 0.69 in the BBR 150 group and 4.42 ± 0.33 in the BBR 300 group (both *p* < 0.001). BBR also partially restored antioxidant-related responses, supporting its regulatory effects on hepatic inflammatory and oxidative stress pathways (Figure 5C).

**Figure 6:**
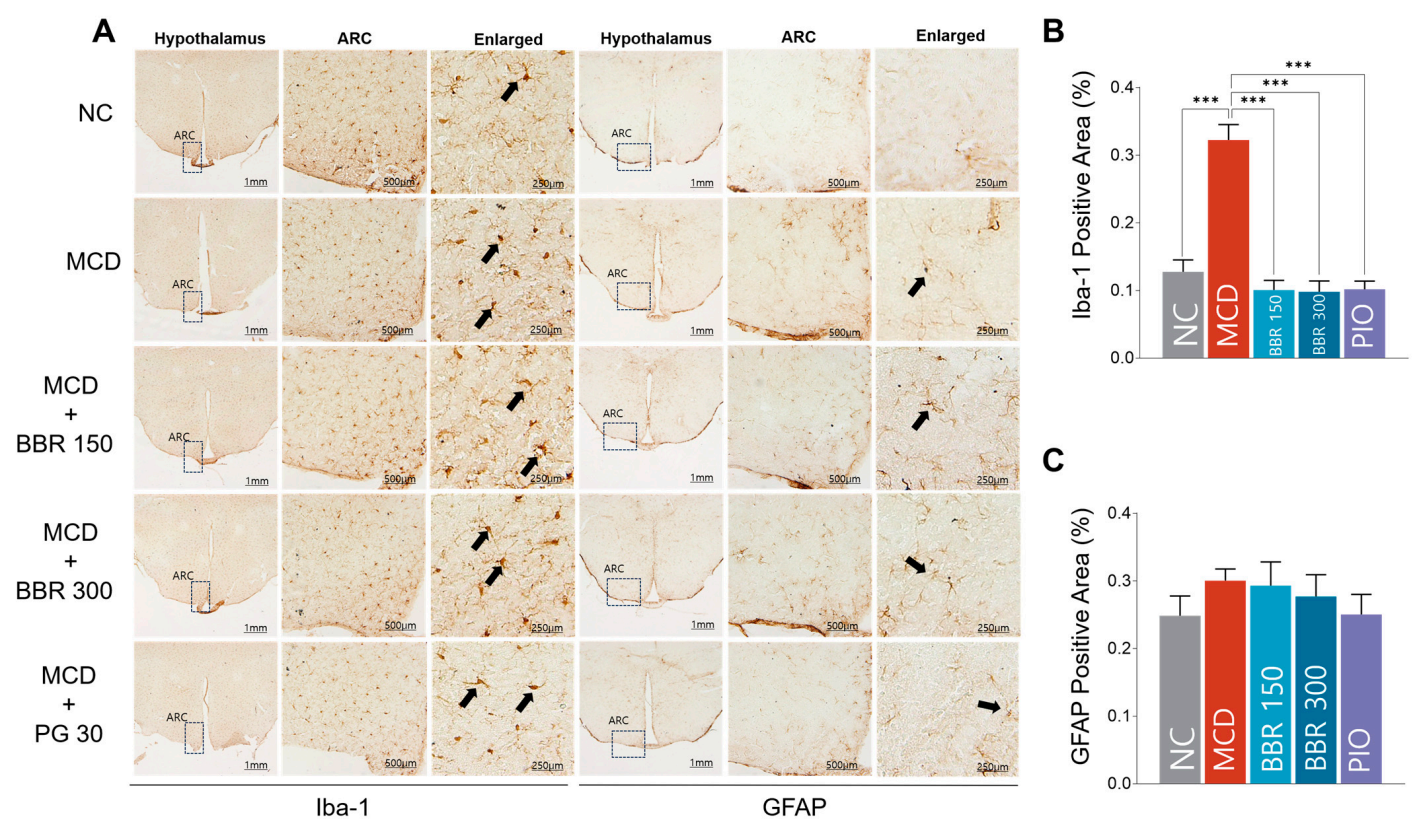
BBR exerts hypothalamic immunomodulatory effects in MCD diet-induced steatohepatitis. (**A**) Representative immunohistochemical images of hypothalamic sections showing microglial (Iba-1) and astrocytic (GFAP) activation. (**B**) Quantitative analysis of Iba-1 (**C**) Quantitative analysis of GFAP. Data are presented as mean ± SEM; *** *p* < 0.001; NC, Normal Chow group; MCD, MCD diet group; BBR 150, MCD diet + Berberine 150 mg/kg/day; BBR 300, MCD diet + Berberine 300 mg/kg/day; PIO, MCD diet + Pioglitazone 30 mg/kg/day. Arrows indicate activated glial cells (scale bars = 1 mm, 500 μm, and 250 μm).

Importantly, immunohistochemical analysis of the hypothalamus revealed marked microglial activation in MCD-fed mice, as demonstrated by increased Iba-1 immunoreactivity compared with the NC group (0.32 ± 0.07 vs. 0.13 ± 0.04, *p* < 0.001). BBR treatment significantly attenuated this increase in a dose-dependent manner, with Iba-1 levels reduced to 0.10 ± 0.03 in the BBR 150 group and 0.09 ± 0.03 in the BBR 300 group compared with the MCD group (both *p* < 0.001) (Figure 6A,B). Pioglitazone also reduced Iba-1 immunoreactivity to a similar extent. In contrast, GFAP immunoreactivity showed only a modest decreasing tendency following treatment, but this change did not reach statistical significance (Figure 6C).

Collectively, these findings demonstrate that BBR reshaped MCD diet-induced inflammatory responses by reducing pro-inflammatory monocyte expansion, hepatic macrophage/Kupffer cell accumulation, and hepatic inflammatory gene expression. Furthermore, BBR attenuated hypothalamic microglial activation, suggesting that its protective effects may extend beyond the liver to modulation of the liver–brain inflammatory axis.

## 3. Discussion

Therapeutic development for metabolic dysfunction-associated steatohepatitis (MASH) remains challenging because disease progression is driven by intertwined metabolic, inflammatory, fibrogenic, and extrahepatic immune mechanisms. In the present study, we identified berberine (BBR) as a multi-level biological modulator that attenuates inflammatory and fibrotic features of MCD- induced MASH by reprogramming hepatic lipid metabolism, immune activation, fibrogenic signaling, and hypothalamic neuroinflammation. By integrating network pharmacology, molecular docking, and in vivo validation, this study delineates a systems-level mechanism through which BBR acts not on a single dominant pathway, but through coordinated regulation of hepatic and neuroimmune transcriptional networks. To our knowledge, this study provides experimental evidence linking BBR-mediated improvement of MCD-induced steatohepatitis with attenuation of hypothalamic microglial activation.

MASH is a multi-stage pathological process involving hepatic lipid accumulation, lipotoxic stress, inflammatory cell recruitment, stellate cell activation, extracellular matrix deposition, and systemic immune dysregulation [17]. In the present network pharmacology workflow, we specifically prioritized lipid metabolism, inflammation, fibrosis, and neuroimmune signaling because these processes represent the principal effector axes of MASH progression and directly correspond to our in vivo experimental readouts. Thus, the computational filtering was intentionally centered on the metabolic–inflammatory–fibrotic axis and its extrahepatic extension to the liver–brain inflammatory pathway, rather than on broad MASH-related terminology alone. Consequently, this attenuation of hepatic injury limited the amplification of inflammatory signaling both locally within the liver and systemically, reducing the propagation of detrimental signals to extrahepatic organs, including the central nervous system.

Mechanistically, BBR suppressed SREBF1-centered lipogenic signaling, which represents a key upstream regulatory axis driving hepatic lipid accumulation and metabolic stress. SREBF1 activation promotes de novo lipogenesis and contributes to hepatocellular lipid overload, thereby amplifying lipotoxic injury and downstream inflammatory signaling [18]. Recent studies have broadened the mechanistic understanding of berberine in MASLD/MASH beyond its direct metabolic and anti-inflammatory actions. Berberine has been shown to modulate the gut–liver axis by increasing Akkermansia muciniphila abundance, enhancing MUC2 expression and mucus-layer integrity, and reducing intestinal permeability, microbial translocation, and hepatic inflammation [19]. At the hepatocellular level, it improves glucose and lipid metabolism by activating PI3K/Akt signaling and suppressing STING-mediated inflammatory responses, with Akt inhibition abolishing these effects and supporting an upstream regulatory role of PI3K/Akt [20]. Berberine also activates the AMPK–SREBP-1c–SCD1 pathway, promoting SREBP-1c phosphorylation and limiting its nuclear activation, thereby reducing SCD1 transcription and hepatic triglyceride synthesis. The functional importance of SCD1 was confirmed by the observation that its knockdown mimicked, whereas its overexpression weakened, the lipid-lowering effects of berberine [21]. The observed reduction in hepatic lipid accumulation following BBR administration suggests that BBR acts during an early pathogenic phase to limit the metabolic stress that initiates and sustains inflammatory and fibrogenic cascades. This effect may involve upstream modulation of AMPK, SIRT, PPARγ, and PI3K/AKT-related signaling pathways, which have been previously implicated in the metabolic actions of BBR. Therefore, BBR appears to function as a metabolic reprogramming cue that suppresses the initiation of hepatocellular injury rather than merely attenuating late-stage inflammatory consequences.

Network pharmacology and docking analyses further revealed that SREBF1, PPARγ, AKT1, and other inflammation- and metabolism-related genes may serve as central nodes mediating the pharmacological activity of BBR. These targets converge at the interface between lipid handling, inflammatory transcription, oxidative stress, and fibrogenic activation, all of which are essential for MASH progression. Although these genes are not exclusive to hepatocytes or immune cells, they function as upstream integrators that coordinate metabolic stress responses and inflammatory amplification across the hepatic microenvironment. Therefore, their identification reflects regulation of the core effector network of MASH rather than a disconnect between the *in silico* prediction and experimental validation.

Consistent with this network-level prediction, BBR markedly attenuated hepatic inflammation and fibrotic remodeling in vivo. The reduction in hepatic inflammatory markers and F4/80^+^ macrophage/Kupffer cell-enriched populations indicates that BBR suppresses local immune activation within the liver. In parallel, the shift in circulating monocyte subsets, characterized by a reduction in Ly6Chi pro-inflammatory monocytes and partial restoration of Ly6Clo monocytes, suggests that BBR reshapes systemic immune responses toward a more resolving phenotype. Given that Ly6Chi monocytes contribute to hepatic inflammation and fibrosis, whereas Ly6Clo monocytes are associated with resolution and tissue repair [22], these findings support the concept that BBR regulates MASH progression through immunometabolic reprogramming rather than through hepatocyte-directed lipid lowering alone.

The present study also revealed that MCD-induced hepatic injury was accompanied by hypothalamic microglial activation, supporting the emerging view that MASH is not a liver-restricted disorder but a systemic inflammatory condition involving liver–brain communication [23]. The attenuation of hypothalamic microglial activation following BBR treatment suggests that improvement of peripheral hepatic inflammation is closely linked to suppression of central neuroimmune responses. Chronic hepatic inflammation may propagate to the central nervous system through circulating cytokines, altered blood–brain barrier integrity, vagal and neuroendocrine signaling, and immune-mediated activation of hypothalamic glial cells [24]. Thus, the observed regulation of hypothalamic microglia provides a mechanistic bridge between hepatic metabolic injury and central inflammatory adaptation. Previous studies have demonstrated that BBR exerts neuroprotective and anti-neuroinflammatory effects in various experimental models through mechanisms involving the suppression of oxidative stress, inhibition of inflammatory signaling, and modulation of microglial activation. Berberine can cross the blood–brain barrier and exert central nervous system effects, although limited brain exposure may restrict its therapeutic efficacy; accordingly, nanocarrier-based and intranasal delivery systems have been explored to improve brain targeting [25]. Across models of Alzheimer’s disease, Parkinson’s disease, and ischemic stroke, berberine suppresses glia-mediated inflammation through the TLR4/MyD88/NF-κB, NLRP3 inflammasome, and MAPK pathways, while activating PI3K/Akt/ERK/Bcl-2 and Nrf2/HO-1 signaling to reduce mitochondrial apoptosis and oxidative stress [26]. It also regulates AMPK/mTOR/Beclin-1-dependent autophagy to facilitate the clearance of amyloid-β and other neurotoxic proteins, attenuates glutamate-mediated excitotoxicity, promotes reparative microglial polarization, and preserves blood–brain barrier integrity partly through MMP-9 inhibition, thereby supporting neuronal survival and function [27]. Previous studies have shown that BBR regulates multiple signaling pathways, including AMPK [28], SIRT3 [29], NF-κB [30], PI3K/AKT [31], and PPARγ signaling [32], primarily in high-fat diet-induced metabolic models. However, such models often incompletely reproduce the inflammatory [33] and fibrotic features [34] observed in progressive MASH. In contrast, the MCD model provides a rapid and robust platform for evaluating hepatic steatosis, inflammation, and fibrosis, although it does not fully reflect obesity-associated insulin resistance [35]. The present study extends previous findings by integrating hepatic metabolic regulation, systemic immune remodeling, fibrogenic suppression, and hypothalamic neuroinflammation into a unified mechanistic framework. This systems-level interpretation distinguishes BBR from conventional single-target therapeutic approaches and supports its potential as a multi-axis modulator in complex metabolic inflammatory diseases.

In brief, BBR attenuated inflammatory and fibrotic features of MCD-induced MASH through coordinated regulation of the hepatic metabolic–immune network and the liver–brain inflammatory axis. In this framework, suppression of SREBF1-driven lipogenesis reduced hepatic lipid accumulation and lipotoxic stress, thereby dampening inflammatory amplification and fibrogenic activation. This hepatic improvement was accompanied by systemic monocyte reprogramming and attenuation of hypothalamic microglial activation, suggesting that BBR enhances disease resolution through distributed regulation of peripheral and central inflammatory states.

Despite these findings, several limitations should be acknowledged. First, the MCD diet model does not fully recapitulate the pathophysiology of human MASH in the context of obesity, insulin resistance, and dyslipidemia. Although this model is useful for inducing rapid hepatic steatosis, inflammation, and fibrosis, future studies using diet-induced obesity, Western diet, or diabetic MASH models are necessary to validate the therapeutic relevance of BBR under metabolically representative conditions. Second, the present study investigated purified BBR rather than a chemically characterized* Coptidis Rhizoma* extract. Therefore, the findings cannot fully represent the pharmacological activity of the crude herbal drug or its multi-constituent interactions. Future studies should compare purified BBR with standardized *Coptidis Rhizoma* extract and include chemical profiling to clarify the contribution of BBR within the traditional herbal matrix. Third, although hypothalamic microglial activation was evaluated, the downstream consequences of liver–brain axis modulation were not assessed. Neurobehavioral outcomes, appetite regulation, energy homeostasis, and cognitive function should be examined in future studies to determine the functional significance of central neuroimmune regulation. Fourth, the central bioavailability of BBR was not directly measured. Although several preclinical studies suggest that berberine can reach the brain, direct evidence of sufficient brain exposure following systemic administration remains limited. Therefore, the beneficial effects observed in the brain may result from both direct central actions and indirect mechanisms, including improvements in systemic metabolism, attenuation of peripheral inflammation, and modulation of the liver–brain axis. Moreover, despite its broad pharmacological activities, the clinical utility of orally administered berberine is limited by poor absorption and permeability, extensive first-pass metabolism, P-glycoprotein-mediated efflux, self-aggregation, and hepatobiliary re-excretion. Structural modification may improve its pharmacokinetic and pharmacological properties, although predicted ADME parameters cannot substitute for experimentally measured bioavailability [36]. In the present study, brain concentrations of berberine were not measured; thus, we cannot determine whether the observed effects were mediated by direct BBB penetration. Finally, the relatively small sample size and the preclinical nature of the study limit the generalizability of the findings. Well-designed clinical and translational studies are required to confirm the efficacy, safety, pharmacokinetics, and mechanistic relevance of BBR in patients with MASH.

## 4. Materials and Methods

### 4.1. Analysis of BBR Using Network Pharmacology

#### 4.1.1. Selection and Physicochemical Structure of BBR

The potential pharmacological targets of BBR were identified using public databases, including SwissTargetPrediction, GeneCards, and related disease–target resources. MASH-related genes were collected using search terms associated with hepatic steatosis, lipid metabolism, inflammation, fibrosis, and neuroimmune signaling. These biological terms were selected because they represent the principal effector axes of MASH progression and directly correspond to the in vivo experimental readouts of the present study. The intersection between predicted BBR targets and MASH-related genes was determined using Venny ver 2.1. A protein–protein interaction network was constructed using the STRING database and imported into Cytoscape software ver 3.10.4 for visualization. Topological analyses were performed to identify hub and core targets based on degree, betweenness, and closeness centralities. Gene Ontology and Kyoto Encyclopedia of Genes and Genomes pathway enrichment analyses were performed, and biological processes or pathways with *p*-values < 0.05 were considered significantly enriched.

#### 4.1.2. Molecular Docking Simulations of Core Targets

Molecular docking analysis was conducted to evaluate the binding affinity between BBR and selected core targets. The three-dimensional structure of BBR was obtained from the PubChem database, whereas crystal structures of target proteins were retrieved from the RCSB Protein Data Bank. Prior to docking, water molecules and co-crystallized ligands were removed using PyMOL ver 3.1. Docking simulations were performed using AutoDock Vina ver. 1.2.7, and ligand–protein interactions were visualized using LigPlot+ ver 1.4 or Discovery Studio Visualizer (version 24.1.0.23298).

### 4.2. In Vivo Study

#### 4.2.1. Preparation of BBR

Berberine chloride hydrate (CAS No. 141433-60-5; catalog no. B0450; purity >98.0% by HPLC) was purchased from Tokyo Chemical Industry Co., Ltd. (TCI, Tokyo, Japan). compound was dissolved or suspended in sterile saline immediately before administration and prepared at the designated concentrations for oral administration. The prepared BBR solution was stored under appropriate conditions and freshly prepared when required to ensure experimental consistency. In the present study, purified BBR was used to evaluate the intrinsic pharmacological activity of this representative isoquinoline alkaloid, rather than the multi-constituent effects of *Coptidis Rhizoma* extract.

#### 4.2.2. Animals and MCD-Induced MASH Model

Six-week-old male C57BL/6J mice were purchased from Central Lab Animals, Inc. (Seoul, Republic of Korea). Animals were housed under controlled environmental conditions with free access to food and water. All animal experiments were performed in accordance with the “Guide for the Care and Use of Laboratory Animals, 8th edition” (National Institutes of Health, 2011 [37]) and the use of laboratory animals was approved by the Institutional Animal Care Committee (KHMC-IACUC 2024-030).

MASH was induced using a methionine- and choline-deficient (MCD) diet. This model was selected because it rapidly induces hepatic steatosis, inflammatory cell infiltration, hepatocellular injury, and fibrotic remodeling, thereby providing a suitable platform for evaluating inflammatory and fibrogenic features of steatohepatitis. Notably, although the MCD diet does not fully reproduce the obesity- and insulin resistance-associated metabolic phenotype of human MASH, it is widely used to investigate hepatic inflammation and fibrosis within a relatively short experimental period.

To ensure ethical standards and animal welfare, animals were monitored throughout the experimental period for body weight changes, food and water intake, coat condition, activity level, and other signs of distress. No animals exhibited severe morbidity requiring early euthanasia. At the end of the experimental period, liver, blood, and hypothalamic tissues were collected for biochemical, histological, immunological, and molecular analyses.

#### 4.2.3. Experimental Design and Drug Administration

The experiment was designed to evaluate the effects of BBR on hepatic metabolic injury, inflammatory activation, fibrogenic remodeling, and liver–brain neuroimmune communication in MCD-induced MASH. Mice were randomly divided into the following groups: (1) normal diet group (NC, vehicle-treated, *n* = 5); (2) MCD diet group (MCD, vehicle-treated, *n* = 5); (3) MCD diet plus low-dose BBR group (150 mg/kg, p.o., *n* = 5); (4) MCD diet plus high-dose BBR group (300 mg/kg, p.o., *n* = 5); and (5) positive comparator group (Pioglitazone, 30 mg/kg, *n* = 5). BBR was administered at doses of 150 mg/kg and 300 mg/kg [38], which were chosen according to previous studies reporting efficacy in mouse models of metabolic disorders and hepatic steatosis. Pioglitazone was administered at a dose of 30 mg/kg as a positive control based on its well-established efficacy in preclinical MASH models [39].

BBR and the positive comparator were administered by oral gavage once daily for 28 days. The low- and high-dose BBR groups were included to assess dose-dependent regulation of hepatic injury and systemic inflammatory responses. The positive comparator group was included to provide a pharmacological reference for evaluating the relative therapeutic activity of BBR in MASH-associated hepatic inflammation and fibrosis.

#### 4.2.4. Tissue Preparation

At the end of the experimental period, mice were anesthetized and sacrificed according to institutional guidelines. Blood samples were collected for serum biochemical analysis. Liver tissues were rapidly excised, rinsed with phosphate-buffered saline (PBS), and divided for histological, molecular, and immunological analyses. Portions of liver tissue were fixed in 10% neutral buffered formalin for histological staining, whereas the remaining tissues were snap-frozen in liquid nitrogen and stored at −80 °C until RNA and protein extraction.

Hypothalamic tissues were carefully dissected to evaluate central neuroimmune responses. The collected hypothalamic samples were either fixed for immunofluorescence analysis or rapidly frozen for molecular analysis. This tissue preparation strategy was designed to align hepatic pathological readouts with systemic immune profiling and central neuroinflammatory assessment.

#### 4.2.5. Serum Biochemical and Hepatic Histological Analysis

Serum alanine aminotransferase (ALT), aspartate aminotransferase (AST), triglyceride, cholesterol, and other metabolic parameters were measured using commercially available assay kits according to the manufacturer’s instructions.

For histological evaluation, fixed liver tissues were dehydrated through a graded ethanol series, cleared in xylene, and embedded in paraffin. Sections were cut at a thickness of 4 μm and stained with hematoxylin and eosin (H&E) to assess hepatic steatosis and inflammatory infiltration. To evaluate lipid accumulation, frozen liver sections were stained with Oil Red O. Fibrotic remodeling was assessed using Masson’s trichrome staining and/or Sirius Red staining. Histological changes were observed using a light microscope (Olympus, Tokyo, Japan), and representative images were obtained for quantitative analysis

#### 4.2.6. Flow Cytometric Analysis of Immune Cell Populations

To assess systemic and hepatic immune remodeling, circulating leukocytes and liver non-parenchymal cells were isolated as previously described with minor modifications. Red blood cells were removed using red blood cell lysis buffer, and the remaining cells were washed with PBS containing fetal bovine serum. Cells were incubated with fluorochrome-conjugated antibodies against monocyte and macrophage markers. For hepatic immune cell analysis, liver tissues were enzymatically digested with collagenase and DNase I to generate single-cell suspensions, which were subsequently filtered and processed similarly for flow cytometric staining. Hepatic cells were stained with antibodies against CD45-APC Cyanine7, F4/80-APC, and CD11b-phycoerythrin Cyanine7, and hepatic F4/80^+^ macrophage/Kupffer cell-enriched populations were defined as CD45^+^F4/80^+^ and CD45^+^F4/80^+^CD11b^+^ populations.

Flow cytometric analysis was performed using a BD Canto flow cytometer (BD Biosciences, San Jose, CA, USA), and data were analyzed using FlowJo software version 10.8.1 (Tree Star Inc., Ashland, OR, USA). Ly6Chi and Ly6Clo monocyte subsets were quantified to determine whether BBR shifted circulating immune responses from a pro-inflammatory phenotype toward a more resolving phenotype. Hepatic F4/80-positive macrophage/Kupffer cell-enriched populations were also analyzed to evaluate local hepatic immune activation.

#### 4.2.7. Immunofluorescence Analysis of Hypothalamic Microglial Activation

To evaluate central neuroinflammatory responses associated with MCD-induced hepatic injury, hypothalamic tissues were subjected to immunofluorescence staining. The brains were cryo-sectioned using a Cryostat (Microsystems AG, Leica, Wetzlar, Germany) with 30 μm thick coronal sections and stored in a cryoprotectant solution, consisting of 0.2 M PB, 25% ethylene glycol, 25% glycerol, and water at 4 °C Sections were then incubated overnight at 4 °C with rabbit anti-ionized calcium-binding adaptor molecule 1 antibody (anti-Iba-1, 1:1000; Wako, Osaka, Japan) and rabbit anti-glial fibrillary acidic protein antibody (anti-GFAP, 1:5000; Neuromics, Edina, MN, USA) to assess microglia and astrocytic activation, respectively, in the hypothalamus. After incubation with appropriate secondary antibodies, immunoreactivity was visualized using 3,3′-diaminobenzidine (DAB, Sigma, St. Louis, MO, USA). Positive staining was semi-quantitatively or quantitatively analyzed using ImageJ software (version 1.53e) by measuring the proportion of immunoreactive area or cell density within defined regions of interest. Microglial activation was assessed based on Iba1-positive cell density, morphological changes, and/or fluorescence intensity. This analysis was performed to determine whether hepatic inflammatory injury was accompanied by hypothalamic neuroimmune activation and whether BBR could attenuate this liver–brain inflammatory response.

All data are presented as mean ± standard error of the mean (SEM). Statistical analyses were performed using GraphPad Prism (version 8.0). Differences among groups were analyzed using one-way analysis of variance (ANOVA) followed by Tukey’s post hoc test for multiple comparisons. A two-tailed *p* value < 0.05 was considered statistically significant. Given the relatively small sample size, the findings should be interpreted as preclinical and hypothesis-generating, and further validation in larger cohorts is warranted.

#### 4.2.8. RNA Extraction and Quantitative Real-Time Reverse Transcription-Polymerase Chain Reaction (qRT-PCR)

Total RNA was extracted from liver and hypothalamic tissues using TRIzol reagent according to the manufacturer’s protocol. RNA concentration and purity were determined using a NanoDrop spectrophotometer (Zymo Research, Irvine, CA, USA). Complementary DNA was synthesized using a reverse transcription kit, and qRT-PCR was performed using SYBR Green-based master mix and a real-time PCR detection system Applied Biosystems, Carlsbad, CA, USA). The expression levels of genes related to lipid metabolism, inflammation, fibrosis, and neuroimmune activation were analyzed. Target genes included *SREBF1*, *FASN*, *TNFα*, *IL1β*, *IL6*, *COL1A1*, *ACTA2*, *TGFB1*, *F4/80*, Iba1, and other markers relevant to hepatic and hypothalamic inflammatory responses. Gene expression was normalized to glyceraldehyde-3-phosphate dehydrogenase (GAPDH), and relative expression levels were calculated using the 2^−ΔΔCt^ method.

#### 4.2.9. Statistical Analysis

All statistical analyses were performed using GraphPad Prism version 8.0 (GraphPad Software Inc., San Diego, CA, USA). Data are expressed as mean ± standard error of the mean (SEM). Before applying parametric tests, normality and homogeneity of variance were evaluated using the Shapiro–Wilk test and Levene’s test, respectively. One-way analysis of variance (ANOVA) followed by Dunnett’s post hoc test was used for comparisons among multiple groups when the data satisfied the assumptions for parametric analysis. A *p*-value less than 0.05 was considered statistically significant. Given the exploratory preclinical design and small sample size, statistical results were interpreted together with effect direction and biological consistency.

## 5. Conclusions

In summary, BBR attenuated inflammatory and fibrotic features of MCD-induced steatohepatitis not only by suppressing SREBF1-centered hepatic lipogenic signaling but also by reprogramming systemic immune responses and hypothalamic neuroimmune activation. This integrative modulation of lipid metabolism, inflammation, fibrosis, and liver–brain immune communication underscores the potential of BBR as a system-level therapeutic candidate for MASH. Although further validation in metabolically representative MASH models and clinical studies is required, the present findings provide a mechanistic basis for the development of BBR as a multi-axis modulator of metabolic inflammatory liver disease.

## Data Availability

The original contributions presented in this study are included in the article. Further inquiries can be directed to the corresponding author.

## Abbreviations

The following abbreviations are used in this manuscript:

ACTA2	Alpha-smooth muscle actin
ALT	Alanine aminotransferase
AST	Aspartate aminotransferase
BBR	Berberine
CAT	Catalase
CCL2	C-C motif chemokine ligand 2
COL3A1	Collagen type III alpha 1 chain
CPT1A	Carnitine palmitoyltransferase 1A
CYP2E1	Cytochrome P450 2E1
FASN	Fatty acid synthase
GFAP	Glial fibrillary acidic protein
GO	Gene Ontology
Iba-1	Ionized calcium-binding adapter molecule 1
IL6	Interleukin 6
KEGG	Kyoto Encyclopedia of Genes and Genomes
Ly6C	Lymphocyte antigen 6 complex, locus C
MASH	Metabolic dysfunction-associated steatohepatitis
MCD	Methionine- and choline-deficient
PPARα	Peroxisome proliferator-activated receptor alpha
SOD2	Superoxide dismutase 2
SREBF1	Sterol regulatory element-binding transcription factor 1
TGFB1	Transforming growth factor beta 1
TIMP1	Tissue inhibitor of metalloproteinase 1
TNF-α	Tumor necrosis factor alpha
